# Synergistic effects of TOR and proteasome pathways on the yeast transcriptome and cell growth

**DOI:** 10.1098/rsob.120137

**Published:** 2013-05

**Authors:** Nianshu Zhang, Zhenzhen Quan, Bharat Rash, Stephen G. Oliver

**Affiliations:** 1 Cambridge Systems Biology Centre and Department of Biochemistry, University of Cambridge, Sanger Building, 80 Tennis Court Road, Cambridge CB2 1GA, UK; 2 Faculty of Life Sciences, The University of Manchester, Michael Smith Building, Oxford Road, Manchester M13 9PT, UK

**Keywords:** proteasome, TORC1, starvation response, Yak1, Rim15, Rpn4

## Abstract

The proteasome has been implicated in gene transcription through a variety of mechanisms. How the proteasome regulates genome-wide transcription in relation to nutrient signalling pathways is largely unknown. Using chemical inhibitors to compromise the functions of the proteasome and/or TORC1, we reveal that the proteasome and TORC1 synergistically promote the expression of *de novo* purine and amino acid biosynthetic genes, and restrict the transcription of those associated with proteolysis, starvation and stress responses. Genetic analysis demonstrates that TORC1 negatively regulates both the Yak1 and Rim15 kinases to modulate starvation-specific gene expression mediated by the Msn2/4 and Gis1 transcription factors. Compromising proteasome function induces starvation-specific gene transcription in exponential-phase cells and abrogates the strict control of such expression by Yak1 and Rim15 in rapamycin-treated cells, confirming that the proteasome functions to ensure stringent control of the starvation response by the TOR pathway. Synergy between the two pathways is also exhibited on cell growth control. Rpn4-dependent upregulation of proteasomal genes and a catalytically competent 20S proteasome are essential for yeast cells to respond to reduced TORC1 activity. These data suggest that the proteasome and the TOR signalling pathway synergistically regulate a significant portion of the genome to coordinate cell growth and starvation response.

## 2. Introduction

Most non-lysosomal/vacuolar protein degradation is carried out by the proteasome in cytosolic and nuclear compartments of eukaryotic cells. Proteasome-mediated degradation, a highly regulated process, can be ubiquitin-dependent or ubiquitin-independent [1,2]. Recently, the 26S proteasome and its subcomplexes have been implicated in the regulation of gene transcription through a variety of mechanisms, including transcription factor (TF) processing and chromatin association (recently reviewed in [3–5]). Processing by the proteasome can restrict the steady-state levels of a TF to limit transcription activation [6–8], convert a TF into a functional state or change its location via limited proteolysis to activate transcription [9–10].

In a number of cases, including Gcn4, and perhaps Gal4 and Ino2/4 [11], proteasome-mediated degradation of these TFs is necessary to stimulate transcription. Inhibiting the proteasome function increases the abundance of these TFs but decreases their transcription activation capabilities. Although the detailed mechanism is not fully understood, it is proposed that these TFs are marked as ‘spent’ after transcription initiation, trapped with the chromatin and unable to stimulate new rounds of transcription. Proteasome-mediated proteolysis destroys such TFs, resets the promoter and allows ‘fresh’ activators to initiate a new round of transcription [5].

Apart from processing TFs directly, the proteasome or its subcomplexes have been shown to associate with chromatin to restrict permissive transcription [12] or to promote transcription initiation, elongation or termination [13–17]. Association of the proteasome or its subcomplexes with chromatin is widespread in the yeast genome [18,19]. Furthermore, the proteasome could function at multiple levels within the same pathway, such as processing TFs into an active state and associating with TF target genes to promote transcription [18].

Although the proteasome has been demonstrated to regulate transcription of many genes, how the proteasome modulates genome-wide transcription in relation to nutrient signalling pathways is largely unknown. Several studies in yeast have examined the transcriptional effects of chemical inhibition of proteasome function [20,21]. Treatment with proteasome inhibitors leads to transcriptional downregulation of genes involved in mating, amino acid metabolism and protein synthesis. Among the genes activated are those implicated in protein degradation and stress response. Previously, we have demonstrated that the post-diauxic shift (PDS) TF Gis1 is subjected to proteasome-mediated proteolysis to downregulate its transcription activation capability [8]. Transcription of PDS genes is moderately activated by the proteasome inhibitor (MG132), significantly induced by rapamycin treatment and hyperactivated by treatment with both drugs, suggesting that the proteasome and TORC1 cooperate to modulate PDS gene transcription. Here, we extend the study to the transcriptome level, and reveal that the proteasome and the TOR signalling pathway synergistically regulate transcription of a significant portion of the genome. Bioinformatic, genetic and phenotypic analyses shed new insights into how the two pathways cooperate in gene transcription, cell growth and starvation response.

## 3. Material and methods

### 3.1. Strains, plasmids and culture conditions

Yeast deletion strains, generated by *Saccharomyces* Genome Deletion Project [22], or decreased abundance by mRNA perturbation (DAmP) strains bearing hypomorphic alleles of essential genes made by Breslow *et al*. [23], were obtained from Open Biosystems. Deletion of *RPN4* in *pdr5Δ*::*kan*MX4 cells was achieved with *HIS3*MX6 marker as described previously [24]. The C-terminus of *RPN4* was similarly tagged with polyhistidine (6-His) at its genomic locus in the *pdr5Δ*::*kan*MX4 cells. Isogenic strains bearing deletions of *msn2Δmsn4Δ* (*msn2/4Δ*); *gis1Δ*; *msn2/4Δgis1Δ*; *rim15Δ*; *yak1Δ* or *rim15Δyak1Δ* were constructed in the *pdr5Δ*::*HIS3*MX6 cells. Overexpression of *MSN2* was achieved by placing the *MSN2* coding sequence under the control of the tetO_7_ promoter in pCM190, as previously described for *GIS1* overexpression [8]. Yeast extract peptone dextrose (YPD) or supplemented minimal medium (SMM) was used throughout the study. A stock solution (1 mg ml^−1^) of rapamycin (Sigma) was made up in 90 per cent (v/v) ethanol and 10 per cent (v/v) Tween-20. MG132 (50 mM; Sigma) was prepared in absolute ethanol. Working concentrations were 200 ng ml^−1^ for rapamycin and 50 µM for MG132 unless otherwise specified.

### 3.2. Microarray analysis

The *pdr5Δ*::*kan*MX4 cells (isogenic to BY4742) were grown to early/mid-exponential phase (OD_600_ ∼ 0.4) in SMM medium. Cultures were split into four flasks, into which the drug vehicle, rapamycin, MG132 or both drugs were added. Samples were taken at 0, 1, 2 and 3 h after treatment. Total RNA was isolated from cultures as described previously [25]. Genome-wide transcription profiling was carried out using the Yeast2 oligonucleotide arrays (Affymetrix Inc.) according to the manufacturer's instructions. Normalization and statistical analyses of the data generated from three biological replicates were performed using Partek Genomics software (http://www.partek.com) as described previously [8,26]. The average correlation coefficients between the triplicate microarray experiments were between 0.983 and 0.994. In compliance with MIAME guidelines, the data from this study have been deposited in the ArrayExpress repository (http://www.ebi.ac.uk/arrayexpress) at the EBI under accession no. E-MTAB-1550.

### 3.3. Analysis of individual transcripts

Total RNA (20 µg) from each sample was used for analyses of individual transcript levels, following the procedures described by Engler-Blum *et al.* [27]. *ACT1* was used as the loading control for all samples. Labelled probes were made using Rediprime II DNA labelling system (GE Healthcare). Phosphoimages were scanned using a Typhoon 9000 imager and analysed using ImageQuant TL software (GE Healthcare). Care was taken to limit the exposure time (typically 8–12 h) to ensure that hybridization signals were not saturated. Hybridization signals from the target transcripts were normalized against that of *ACT1* for each sample. The levels of the transcripts in the wild-type cells at time 0 h of drug treatment were set to the arbitrary unit 1.

### 3.4. Western analysis

Anti-myc (Sigma) and anti-tubulin (Cancer Research, UK) antibodies were used in Western analysis to detect the levels of Rpn4-myc and tubulin, respectively, following the protocol described previously [8].

### 3.5. *GIS1* and *MSN2* overexpression assays

Yeast transformants bearing the empty vector (pCM190) or the overexpression plasmid (tetO_7_-*GIS1* or tetO_7_-*MSN2*) were grown in SMM medium under repressive conditions (plus 20 µg ml^−1^ of doxycycline) to mid-exponential phase. In order to determine the toxic effects of *GIS1* or *MSN2* overexpression on cell growth, cells were harvested, washed and resuspended in water to the same density, and spotted in serial dilutions (sevenfold) on SMM medium plates containing 20 µg ml^−1^ of doxycycline (Dox+) or no doxycycline (Dox−). Cells grown on glucose (2%) were incubated at 30°C for 2 days, and those grown on ethanol (2% v/v) and glycerol (1% v/v) for 7 days.

### 3.6. Determination of growth rates of proteasomal mutants

Continuous monitoring of cell growth in quadruplicate was carried out using a plate reader (BMG Biotech). To determine the effects of a drug on cell growth, the doubling time of cells grown in medium containing the drug was normalized against that of the same cells grown in the presence of the drug vehicle. Working concentrations were 50 ng ml^−1^ for rapamycin and 12.5 µM for MG132.

## 4. Results

### 4.1. TORC1 and proteasome synergistically regulate the transcription of a significant portion of the genome

**Figure 1.**
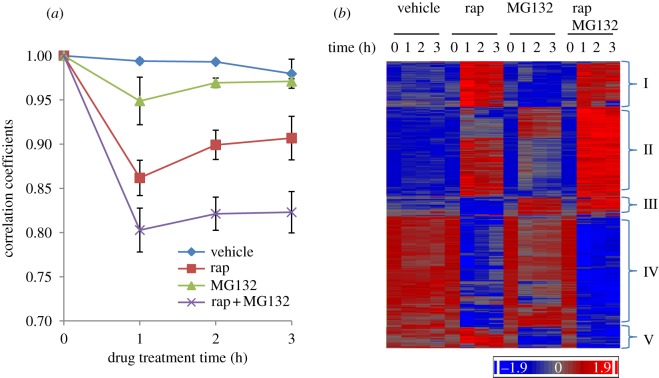
(*a*) Change of correlation coefficients of the transcriptome during drug treatment. These coefficients were calculated between the initial sample and those 1, 2 and 3 h after introduction of the drug. The mean and s.d. were calculated from biological triplicates. (*b*) Hierarchical clustering of genes whose mean transcript levels were changed more than 1.5-fold by drug treatment.

Our previous study [8] indicated that PDS gene transcription mediated by the Gis1 TF is coordinately modulated by the functions of the proteasome and TORC1. To find the extent to which the proteasome and TORC1 cooperate with regulate gene transcription, we treated exponentially growing *pdr5*Δ cells with the drug vehicle, rapamycin (TORC1 inhibitor), MG132 (the proteasome inhibitor) or both drugs. Samples from biological triplicates were taken at 0, 1, 2 and 3 h post-treatment and microarray experiments carried out using Yeast2 arrays. Transcriptome data analysis was performed using Partek Genomics software, and the results are summarized in electronic supplementary material S1. Comparison of transcriptome data at time 0 with those from subsequent time points revealed that treatment with the drug vehicle did not cause significant changes of whole-genome transcription (figure 1*a*). Genome-wide transcription was more significantly altered in cells treated with rapamycin than in cells treated with MG132. Addition of both rapamycin and MG132 triggered a more dramatic change in transcriptome than either drug alone (figure 1*a*).

Out of the 5716 genes detectable with Yeast2 chips, the transcript levels of 3220 open reading frames (ORFs) are changed more than 1.5-fold (*p* < 0.01) by treatment with rapamycin and/or MG132. Among them, the transcription of 1028 ORFs is regulated more than 1.5-fold by MG132 treatment, contrasting with those of 2565 ORFs similarly altered in rapamycin-treated cells. Comparison of our data with other recent studies revealed that around 70 per cent of the rapamycin-induced genes and 60 per cent of the rapamycin-repressed ORFs were similarly regulated by rapamycin treatment of yeast cells of a different genetic background. By contrast, among the ORFs significantly regulated by MG132 treatment, 42.5 per cent of the upregulated and only 14.5 per cent of the downregulated genes were seen to overlap with those revealed by Dembla-Rajpal *et al*. [20]. Poorer overlapping between the MG132-repressed gene sets was possibly due to different methodologies used to interrogate the transcriptome in the two studies (Affymetrix genechips versus gene filters by Dembla-Rajpal *et al*.). An approximately equal number of genes was shown up- or downregulated by MG132 treatment in our study, whereas for Dembla-Rajpal *et al*. [20] the number of the downregulated ORFs was about one-third of that of the upregulated genes in cells treated with MG132 for 120 min.

Agglomerative hierarchical clustering of the 3220 regulated genes revealed five major clusters (figure 1*b*). The majority of these genes fall into two clusters: II and IV. Transcription of cluster II genes is activated by rapamycin or MG132 treatment and shows an even greater increase when cells are treated with both drugs. By contrast, the transcript levels of cluster IV genes are significantly decreased in rapamycin-treated cells, and either moderately reduced or barely changed by MG132 treatment. Addition of both drugs led to a more profound decrease in their expression than either drug alone; this was especially true after prolonged drug treatment (figure 1*b*). Expression of cluster I genes seems to be predominantly activated by rapamycin treatment. Opposite effects of rapamycin and MG132 on transcription are observed in two small clusters (III and V). These data suggest that, despite their independent roles in transcription regulation, TORC1 and the proteasome function synergistically to regulate a significant portion of the yeast transcriptome.

**Figure 2.**
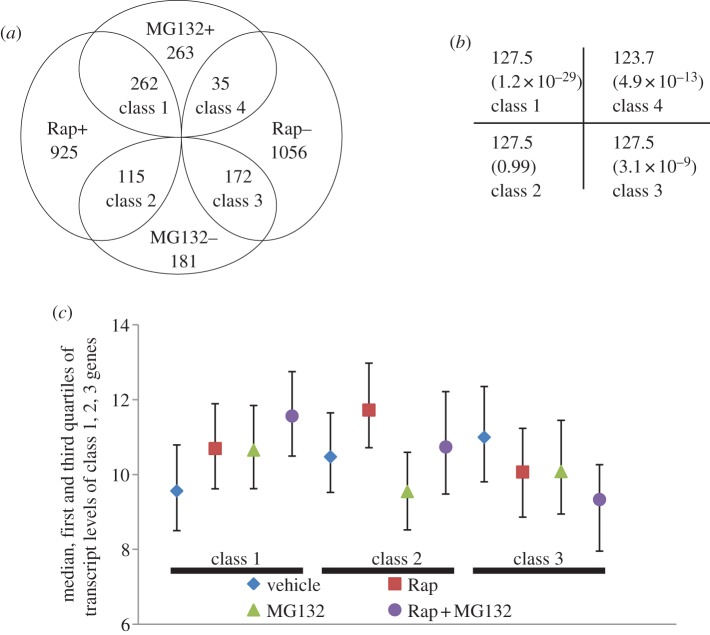
(*a*) The number of genes whose mean transcript level changed more than 1.5-fold in response to rapamycin (Rap) or MG132. ‘+’ and ‘−' denotes up- and downregulation due to drug treatment, respectively. (*b*) The expected number and *p*-values displayed for each class of overlapping genes in (*a*) was revealed by chi-squared test. (*c*) The median, first and third quartiles of the relative transcript levels for each class of genes revealed in (*a*). The data represent mean values for samples taken 1 h after drug or vehicle treatment.

The ORFs that were regulated more than 1.5-fold by both drug treatments were indentified (figure 2*a*; electronic supplementary material S2). The expected number of each class of overlapping genes and their *p*-values (shown in brackets) were calculated by chi-squared test (figure 2*b*). Significantly over-represented were those genes whose transcription was induced (Rap+/MG132+, class 1) or repressed (Rap−/MG132−, class 3) by treatment with either drug. Transcript levels of these genes were more profoundly increased (class 1) or decreased (class 3) in cells treated with both drugs (figure 2*c*). Rapamycin-induced transcription of a number of genes was downregulated by treatment with the proteasome inhibitor (Rap+/MG132−, class 2; figure 2*a,c*). The actual number of genes in this category is, however, very close to the expected value (figure 2*b*). Strikingly, significantly under-represented are those genes whose transcription was repressed by TORC1 inhibition but induced by proteasome inhibition (Rap−/MG132+, class 4; figure 2*a*,*b*). Genes whose transcription is downregulated when TORC1 function is inhibited are inferred to be under the positive control of the complex in nutrient-sufficient conditions [29]. By contrast, genes whose transcription is activated in the presence of the proteasome inhibitor can be inferred to be under the negative control of the proteasome. These data suggested that the proteasome and TORC1 pathways do not tend to act antagonistically to regulate gene transcription (class 4) in nutrient-replete cells and that the two pathways function homo-directionally to modulate the transcription of a significant number of genes in the genome (classes 1 and 3).

### 4.2. Motifs targeted by a number of transcription factors are enriched in the promoter regions of genes co-regulated by proteasome and TORC1

Gene ontology analysis of the class 1 genes (figure 2*a*) revealed that they fall into two major functional categories: proteolysis (*p*-value 1.3 × 10^−9^) and response to stress (*p*-value 1.1 × 10^−6^). The former category was made up of genes implicated in the ubiquitin–proteasome system (UPS; 27 genes) and autophagy/vacuolar function (10 genes). The latter category consisted of those genes involved in starvation and stress response (53 genes). Analysis of the promoter sequences of these ORFs revealed several enriched motifs (*p* < 0.0001), which resembled the different consensus sequences targeted by a number of TFs, including Rpn4, Msn2/Msn4 (Msn2/4), Gis1 and Hsf1 (table 1). Each of these TFs was also shown to regulate the expression of at least 10 per cent of the class 1 genes by YEASTRACT [30].

**Table 1.** Enriched motifs in the promoters of co-regulated genes by TORC1 and proteasome. Question marks denote ‘yet to be identified’.

category	motifs	*p*-value	TF	consensus
Rap + MG132 + (class 1)	GTGGCAAA	6.4 × 10^−16^	Rpn4	GGTGGCAA
	GGTGGCAA	1.2 × 10^−13^	Rpn4	
	AGGGG	1.6 × 10^−13^	Msn2/Msn4	AGGGG
	CGCCAC	1.2 × 10^−8^	?	?
	TTCTAGAA	4.7 × 10^−7^	Hsf1	TTCNNGAA
	CCGCCA	1.5 × 10^−6^	?	?
	AAGGGAT	3.6 × 10^−5^	Gis1	TWAGGGAT
Rap + MG132-(class 2)	CTTATC	1.3 × 10^−11^	Gln3	CTTATC
			Gat1	CTTATC
Rap-MG132-(class 3)	CGCGTC	4.5 × 10^−8^	?	?
	TGACTC	7.0 × 10^−7^	Bas1	TGACTC
			Gcn4	TGASTCA
	ACGCGT	1.1 × 10^−6^	Mbp1	ACGCGT
	CGCGAA	3.0 × 10^−6^	?	?

**Figure 3.**
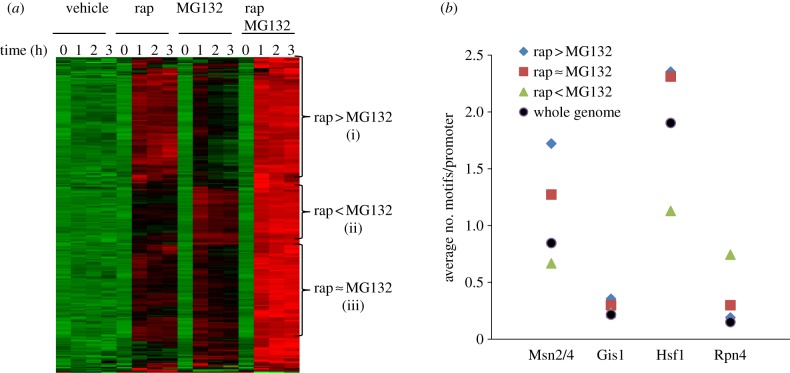
(*a*) Hierarchical clustering of genes significantly upregulated by either rapamycin (rap) or MG132. The means from biological triplicates were used for clustering. Three groups of genes were represented by (i) rap > MG132 for those more strongly activated by rap than by MG132, (ii) rap < MG132 for those less significantly activated by rap than by MG132 and (iii) rap ≈ MG132 for those more or less equally upregulated by either drug. (*b*) The average number of Msn2/4, Gis1, Hsf1 and Rpn4 motifs in the promoter regions of the three groups of genes. The average number of these motifs in the whole genome is also included for comparison.

Class 1 genes can be further divided into three subcategories: (i) more significantly activated by rapamycin than by MG132; (ii) more highly activated by MG132 than by rapamycin; and (iii) more or less equally activated by either drug (see figure 3*a*; electronic supplementary material S3). Category (i) genes were enriched with Msn2/4 and Hsf1 motifs in their promoter sequences (figure 3*b*). Conversely, the genes in category (ii) were predominantly involved in the UPS (see electronic supplementary material S3), with the Rpn4 motif over-represented and the motifs targeted by Msn2/4 and Hsf1 under-represented in their promoter regions (figure 3*b*). Rpn4 is both a target and the activator of the 26S proteasome [31]. Msn2 is degraded by the proteasome in the nucleus under stress conditions [7], and Gis1 is subjected to proteasome-mediated limited proteolysis to downregulate its transcription activation capacity [8]. Rapamycin-induced gene expression mediated by Msn2 and Gis1 is strictly regulated by the TORC1-negatively controlled Rim15 kinase [8,32,33]. These results indicated that the functions of TORC1 and the proteasome converge on a number of TFs to keep starvation- and stress-induced gene transcription in control (see also §§4.3–4.5). Their relative roles in regulating transcription may depend on a variety of promoter contexts (figure 3).

**Figure 4.**
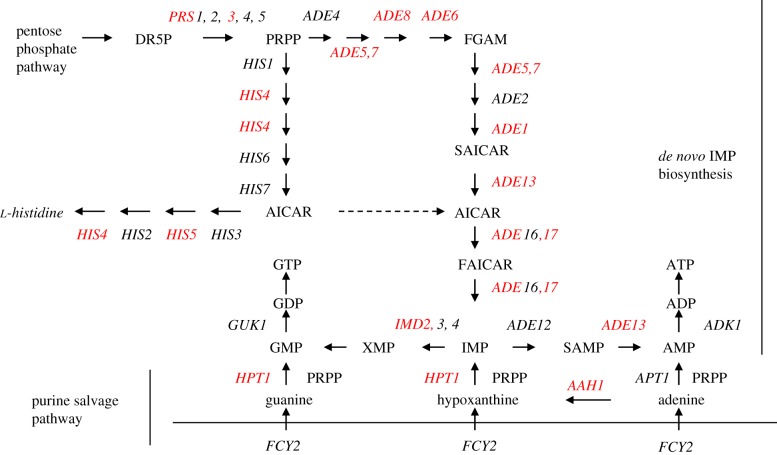
Genes involved in *de novo* IMP biosynthesis and purine salvage pathways were downregulated (in red) by treatment with rapamycin or MG132.

Significantly enriched among the genes downregulated by treatment with rapamycin or MG132 (class 3, figure 2*a*) are those involved in the biosynthesis of cellular nitrogen compounds (*p*-value, 2.1 × 10^−10^), especially *de novo* IMP biosynthesis and amine metabolic process. Purine nucleotides are generated through the purine biosynthesis pathway, using phosphoribosyl pyrophosphate from the pentose phosphate pathway or 5-aminoimidazole-4-carboxamide-1-β-D-ribofuranoside (AICAR) diverted from the histidine biosynthetic pathway as substrates. The alternate pathway for purine nucleotide formation is the purine salvage pathway, using purine bases or nucleosides from the environment. Treatment with either rapamycin or MG132 led to transcriptional repression of many *ADE* genes and a number of genes involved in purine salvage (labelled red in figure 4) and in one-carbon metabolism (*SHM2*, *MTD1*, *GCV1* and *GCV2*). Concurrently repressed were amino acid biosynthetic genes (*ARG1*, *ARG3*, *ARG5,6*, *ARG8*, *ARO3*, *ARO4*, *LEU1*, *LEU2*, *LEU9*, *ILV3*, *MET3*, *MET14* and *SAM2*), ribosomal protein genes (*RPL6B*, *RPS7B*, *RPL9A*, *RPS11B*, *RPL12A*, *RPS14B* and *RPS28B*) and a number of genes implicated in cell cycle regulation (*ACM1*, *ALK2*, *CLB1*, *CLB6*, *CLN1*, *GIC1*, *NRM1*, *PCL1*, *PCL2*, *TOS1*, *TOS2*, *TOS4*, *SHE1*, *SIM1*, *SWE1*, *YHP1* and *YOX1*). The most over-represented 6-mer in the promoter regions of this class of ORFs was CGCGTC (table 1), but there is no TF that is known to target this motif (http://www.yeastract.com/consensuslist.php). The other significantly enriched sequences, TGACTC and ACTGCT (table 1), were targeted by the Bas1 and Mbp1 TFs, respectively [34–36]. The Bas1 target motif is also similar to the Gcn4-bound element (TGASTCA) [37,38]. Gcn4 is a master regulator of gene expression during amino acid starvation, activating the transcription of more than 500 genes involved in amino acid biosynthesis and purine metabolism [39]. Staschke *et al*. [28] demonstrated that both Gcn4 and Gln3 are major effectors of the TOR pathway, with each of them inducing and repressing the transcription of a large number of genes during rapamycin treatment. The steady-state level of Gcn4 is subject to translational regulation by TORC1 [40] and proteasome-mediated degradation [41]. Inhibiting the proteasome function, however, increased the level of the Gcn4 protein but reduced the expression of genes activated by Gcn4 in cells grown in *minimal* medium or starved for amino acids, indicating that degradation of Gcn4 by the proteasome is necessary to stimulate the basal and induced transcription of Gcn4-activated genes [11]. These data suggest that the functions of the proteasome and TORC1 may converge on Gcn4 to regulate the expression of a group of anabolic genes essential to cell growth. This hypothesis needs to be further verified experimentally.

Interestingly, among the genes activated by TORC1 inhibition but repressed by MG132 treatment (class 2, figure 2*a*), ‘amine metabolic process’ (*p*-value: 9.9 × 10^−7^) was also the major enriched functional category, including those whose transcription is sensitive to nitrogen catabolite repression (*CAR1*, *CAR2*, *DUR1,2*, *DUR3*, *DAL3*, *GLT1*, *MEP1* and *MEP2*) or elicited upon starvation for amino acids (*ALT1*, *GAP1*, *DIP5*, *LYS14*, *MET28*, *SAM3*, *SER3* and *SUL1*). The other over-represented functional category was ‘response to pheromone’ (*p* = 0.0005), especially ORFs involved in conjugation with cellular fusion (*FUS1*, *FIG2*, *AFR1*, *MFA1*, *DSE1*, *SAG1*, *FUS2*, *PRM1*, *AGA1* and *RRI2*). The most enriched motif in the promoters of this group of genes was CTTATC (table 1), the consensus sequence targeted by the rapamycin-activated TFs, Gln3 and Gat1 [42]. Deletion of *GCN4* elevates the basal level of nitrogen catabolite repression (NCR)-sensitive genes in cells grown on nitrogen-rich sources [43], and causes hyperactivation of these genes in rapamycin-treated cells [28,43], indicating that Gcn4 functions to repress the transcription of the NCR-sensitive genes. However, the Gcn4 target motifs were not enriched in the promoter sequences of this class of genes (table 1), suggesting that Gcn4 may repress their transcription indirectly. In this regard, Gcn4, in cells subjected to amino acid starvation, was previously shown to bind Rap1, leading to the inhibition of Esa1 recruitment to the promoters of ribosomal protein genes, and ultimately their transcriptional repression [44].

### 4.3. TORC1 and proteasome converge on Msn2/4 and Gis1 to regulate the starvation-induced stress response

**Figure 5.**
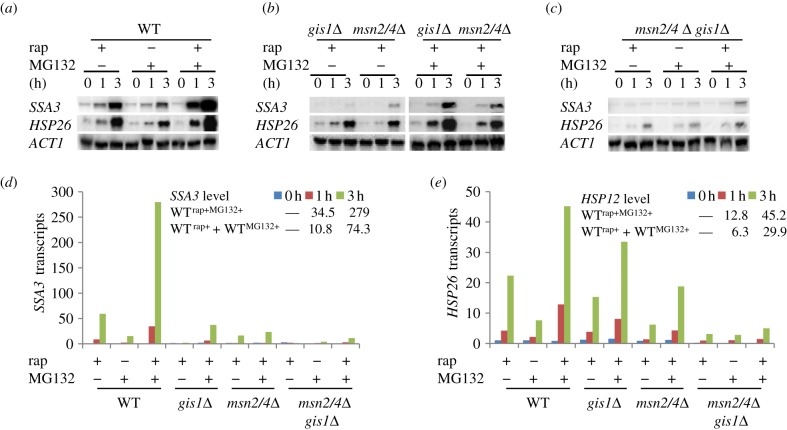
(*a*–*c*) *SSA3* and *HSP26* transcripts detected in WT (*a*), *gis1*Δ and *msn2/4*Δ (*b*), and *gis1*Δ*msn2/4*Δ (*c*) cells treated with MG132 and/or rapamycin. (*d,e*) Normalized transcript levels of *SSA3* (*d*) and *HSP26* (*e*) to that of *ACT1* in WT and mutant cells. The value at time 0 of treatment with rapamycin in WT cells was set to an arbitrary unit of 1.

To further confirm that the proteasome and TOR pathways converge on the TFs (table 1) to control starvation-induced gene expression (class 1, figure 2*a*), we constructed an *msn2Δmsn4Δ* (*msn2/4Δ*) double and an *msn2/4Δgis1Δ* triple mutant in the *pdr5Δ* deletion background. The transcript levels of two class 1 genes, *SSA3* (a PDS gene) and *HSP26* (a STRE gene), were assayed in early exponential-phase cells treated with rapamycin and/or MG132 using Northern analysis. The levels of *SSA3* and *HSP26*, after being normalized to that of *ACT1* (the loading control), are displayed in figure 5*d* (for *SSA3*) and figure 5*e* (for *HSP26*). Rapamycin-induced transcription of *HSP26* and *SSA3* seen in WT cells (lanes 1–3, figure 5*a*) was significantly reduced in the *gis1*Δ or *msn2/4*Δ deletion cells (figure 5*b*, left) and nearly abolished in the *gis1*Δ*msn2/4*Δ triple mutants (lanes 1–3, figure 5*c*). When compared with that seen in rapamycin-treated cells, the transcription of *SSA3* and *HSP26* was moderately induced in WT (*pdr5Δ*) cells treated with the proteasome inhibitor (lanes 4–6, figure 5*a*). This moderate induction was also dramatically reduced in the *gis1*Δ*msn2/4*Δ triple mutants treated with MG132 (lanes 4–6, figure 5*c*). Hyperactivation of *SSA3* and *HSP26* was observed when the WT cells were treated with both drugs (lanes 7–9, figure 5*a*). Such hyperactivation was reduced in either *gis1*Δ or *msn2/4*Δ cells (figure 5*b*, right) and more dramatically decreased in the *gis1*Δ*msn2/4*Δ triple mutants similarly treated (lanes 7–9, figure 5*c*). These data confirmed that the effects of the proteasome and TORC1 on STRE and PDS gene transcription are largely mediated via the Msn2/4 and Gis1 TFs. Furthermore, based on the quantification of *SSA3* and *HSP26* transcripts (figure 5*d* and 5*e*, respectively), we found that the fold-change of either transcript in cells treated with both rapamycin and MG132 is greater than the sum of those in cells treated with either drug (*p* < 0.01). This is true for both time points taken following drug treatment (1 and 3 h), thus confirming that TORC1 and the proteasome synergistically restrict the expression of starvation-specific transcription.

### 4.4. Inhibition of the proteasome function abolished the stringent control of Msn2/4- and Gis1-dependent transcription by both Rim15 and Yak1 kinases

**Figure 6.**
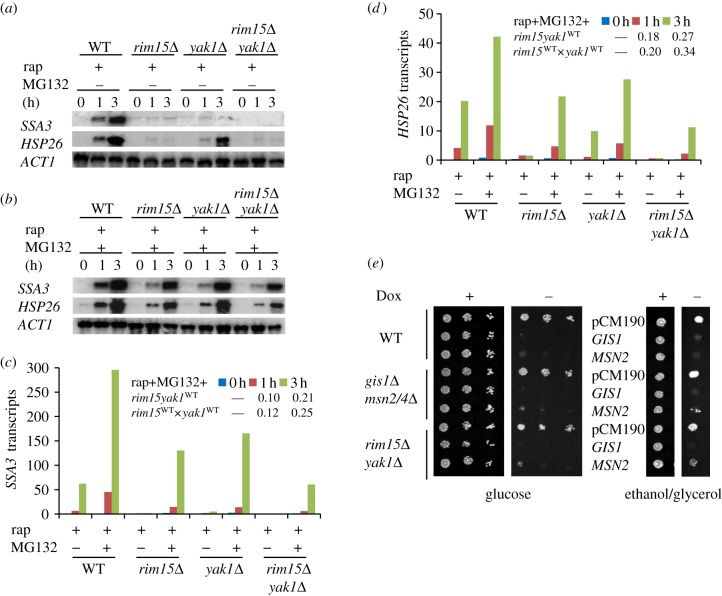
(*a,b*) *SSA3* and *HSP26* transcripts detected in WT (BY4742*pdr5*Δ), *rim15*Δ, *yak1*Δ and *rim15*Δ*yak1*Δ cells treated (*a*) with rapamycin or (*b*) with both rapamycin and MG132. (*c*,*d*) Normalized transcript levels of (*c*) *SSA3* and (*d*) *HSP26* to that of *ACT1* and their level in WT cells at time 0 of treatment with rapamycin was set to an arbitrary value of 1. (*e*) Toxicity of *GIS1* or *MSN2* overexpression to cell growth on glucose (left) or ethanol/glycerol (right). *GIS1* or *MSN2* controlled by the tetO_7_ promoter is switched on in the absence of doxycycline (Dox−) and off in the presence of the drug (+, 20 µg ml^−1^).

Rapamycin-induced transcription mediated by Msn2/4 and Gis1 was previously shown to be strictly dependent on the Rim15 kinase [8,32,33]. Zhang & Oliver [8] have demonstrated that the strict control of *SSA3* transcription by Rim15 in TORC1-inhibited cells was abolished when the function of the proteasome was compromised (see also figure 6*a*,*b*). Similar to *SSA3*, the level of *HSP26* transcripts, which were barely detectable in rapamycin-treated *rim15Δ* cells (figure 6*a*), was significantly increased by concurrent treatment with both rapamycin and MG132 (figure 6*b*), confirming that stringent control of STRE and PDS gene transcription by Rim15 requires the function of the proteasome. These data also suggest that other regulators that are negatively controlled by TORC1 may promote STRE and PDS gene expression via pathways that are parallel or compensatory to that of Rim15. Because the localization of the Yak1 kinase to the nucleus is negatively controlled by TORC1 [45] and Yak1 is necessary for Msn2-mediated transcription in response to glucose starvation [46], we tested the hypothesis that Yak1 is one of the other regulators activating STRE/PDS gene transcription in TORC1-inhibited cells. As shown in figure 6*a*, rapamycin-induced *HSP26* and *SSA3* transcription seen in wild-type cells was dramatically reduced (for *HSP26*) or nearly abolished (for *SSA3*) in the *yak1*Δ deletion cells. Similar to that observed in the *rim15Δ* cells, concurrent treatment with both drugs triggered a significant increase in both transcripts in the *yak1Δ* deletion cells (figure 6*b*). The degree of transcriptional activation, however, is lower in the *yak1*Δ or *rim15*Δ cells than that seen in the WT cells (figure 6*c*,*d*).

To find the relationship between Rim15 and Yak1, a *rim15*Δ*yak1*Δ double mutant was constructed in the *pdr5Δ* deletion background. Transcription activation of *HSP26* and *SSA3* by rapamycin treatment was completely abolished in the *rim15*Δ*yak1*Δ double mutants, as was the case in the *rim15Δ* single mutant (figure 6*a*). Additional MG132 treatment induced the transcription of both genes in the *rim15*Δ*yak1*Δ double mutants to a lesser extent than that seen in the *rim15*Δ or *yak1*Δ single mutants (figure 6*b*). At both post-treatment time points (1 and 3 h), the relative level of *SSA3* or *HSP26* in the *rim15*Δ*yak1*Δ double mutants, when compared with that in the wild-type cells (*rim15yak1*^WT^), was close to the product of its relative levels in the *rim15*Δ and *yak1Δ* single mutants (*rim15*^WT^ × *yak1*^WT^; figure 6*c*,*d*). This implies that, in TORC1-inhibited cells, Yak1 and Rim15 may promote STRE/PDS gene transcription in parallel pathways. Although much reduced, significant amounts of *HSP26* and *SSA3* transcripts were detected in the *rim15*Δ*yak1*Δ cells treated with both drugs (figure 6*b*), suggesting the presence of yet-to-be-identified regulators acting together with Rim15 and Yak1 to support STRE/PDS gene expression. These observations indicated that upon TORC1 inhibition, multiple regulators, including Yak1 and Rim15, are activated or de-repressed to promote STRE/PDS gene transcription. The function of the proteasome, by limiting the abundance of the Msn2 and Gis1 TFs [7,8], is essential to restrict STRE/PDS gene transcription in exponential-phase cells (figure 5) and to ensure strict control of such transcription by the TORC1-negatively controlled regulators (figure 6).

To further characterize the physiological implications of the proteasome-mediated degradation of Msn2 and Gis1, *GIS1* or *MSN2* was over-expressed under the control of the tetO promoter. Previous studies have demonstrated that overexpression of *MSN2* or *GIS1* is toxic to growth [7,8,47]. Overexpression of *MSN2* or *GIS1* also resulted in growth arrest of *gis1*Δ*msn2/4*Δ and *rim15*Δ*yak1*Δ mutant cells grown on glucose (figure 6*d*, left). Similar phenotypes were observed when cells were grown on ethanol/glycerol (figure 6*d*, right), although cells overexpressing *GIS1* displayed a more severe growth defect than those overexpressing *MSN2*, especially in the *gis1*Δ*msn2/4*Δ and *rim15*Δ*yak1*Δ mutants (figure 6*d*, left and right). These data suggest that proteasome-mediated proteolysis of the starvation-specific TFs (Msn2 and Gis1) ensures not only optimal cell growth but also proper transition from exponential growth to the stationary phase.

### 4.5. Transcriptional upregulation of genes coding for proteasomal subunits is mediated by Rpn4 in TORC1-inhibited cells

**Figure 7.**
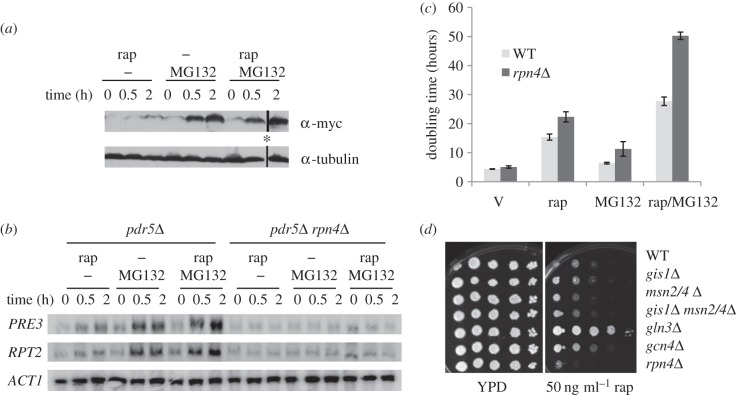
(*a*) The Rpn4 protein level in WT (*pdr5*Δ) cells treated with rapamycin (rap) and/or MG132. Endogenous *RPN4* was tagged with myc in WT (*pdr5*Δ) cells. Mid-exponential cells were treated with rap (200 ng ml^−1^) and/or MG132 (50 µM) for 0, 0.5 and 2 h. *Note that, in the original SDS-PAGE gels, the protein sample representing 2 h of treatment with both drugs was loaded into the well adjacent to the sample denoting 0 h of treatment with rapamycin. (*b*) *PRE3* and *RPT2* transcript levels in WT (*pdr5*Δ) and *rpn4*Δ deletion cells treated with rap and/or MG132 for 0, 0.5 and 2 h. (*c*) The doubling time of WT (*pdr5*Δ) and *rpn4*Δ deletion cells grown in SMM medium containing drug vehicle (V), rap (50 ng ml^−1^), MG132 (12.5 µM) or both. (*d*) Rapamycin hypersensitivity of *rpn4*Δ deletion cells in YPD medium.

The enrichment of the ‘proteolysis’ functional category (figure 2*a*) and the Rpn4 motif (table 1) in the class 1 genes suggested that rapamycin- or MG132-induced transcription of genes encoding proteasomal subunits is mediated by Rpn4. To confirm this, the endogenous *RPN4* reading frame was tagged with myc at its C-terminus and the steady-state level of Rpn4 assayed in cells treated with either or both drugs. As shown in figure 7*a*, the level of Rpn4 was only marginally increased in rapamycin-treated cells and significantly elevated in MG132-treated cells. Treatment with both drugs triggered a slightly more dramatic increase in Rpn4 than MG132 alone, especially at 2 h post-treatment. Correspondingly, the transcription of two proteasomal genes, *PRE3* and *RPT2*, was moderately upregulated in rapamycin-treated cells, significantly activated in MG132-treated cells and more dramatically activated by treatment with both drugs (again more evident at 2 h post-treatment; figure 7*b*). Transcriptional activation of *PRE3* and *RPT2* was abolished in the *rpn4Δ* deletion cells in all treatment conditions (figure 7*b*), confirming that the transcriptional activation of proteasomal genes in TORC1-inhibited cells is mediated through Rpn4, which is similar to the situation seen in MG132-treated cells. To discover the physiological significance of this regulation, wild-type and *rpn4Δ* cells were grown in SMM medium containing sublethal concentrations of rapamycin (50 ng ml^−1^) and/or MG132 (12.5 µM). As demonstrated in figure 7*c*, the doubling time of the wild-type cells is significantly extended by rapamycin treatment, moderately increased by MG132 treatment and greatly extended in the presence of both drugs. The *rpn4Δ* deletant, when treated with the drug vehicle, exhibited marginally slower growth (approx. 15%) than WT cells similarly treated. This slow-growth phenotype was more pronounced in the presence of either or both drugs (figure 7*c*). These results indicate that Rpn4-mediated regulation of proteasomal genes and the function of the proteasome are both necessary for cells to adapt to conditions where TORC1 activity is reduced. Similarly, in comparison with WT cells, the *rpn4Δ* deletant displayed enhanced rapamycin sensitivity when grown in rich medium, as opposed to reduced rapamycin sensitivity shown by the *gln3Δ* or *gcn4Δ* mutants (figure 7*d*). Deletion of *MSN2/4* and/or *GIS1* did not significantly impact on cell growth in the presence of rapamycin (figure 7*d*), further highlighting the importance of Rpn4-dependent regulation of proteasome abundance in response to compromised TORC1 function.

### 4.6. The core functions of the 20S proteasome is essential for yeast cells to respond to compromised TORC1 activity

**Figure 8.**
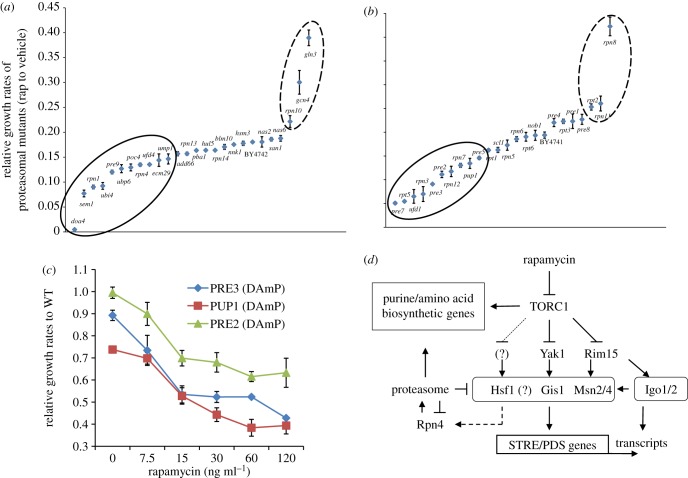
(*a*,*b*) Relative sensitivity of proteasomal mutants to mild inhibition of TORC1. (*a*) Deletion mutants of non-essential genes encoding UPS components and (*b*) DAmP mutants of essential proteasomal genes were grown in quadruplicates in YPD medium containing 50 ng ml^−1^ of rapamycin or drug vehicle. Relative sensitivity was calculated by normalizing mutant cells' growth rate in the presence of rapamycin to that in the presence of the drug vehicle. (*c*) Relative growth rates of the three catalytically compromised DAmP mutants in response to increasing rapamycin concentrations. (*d*) The working model. STRE/PDS gene expression, mediated by Msn2/4, Gis1 and possibly Hsf1, is controlled by the proteasome and multiple TORC1-negatively regulated proteins, Rim15, Yak1 and others. The arrows and bars denote positive and negative controls, respectively. The question marks and dashed lines refer to unconfirmed or unidentified nodes and regulatory connections.

To find out what aspects of proteasome function are required for yeast cells to cope with compromised TORC1 activity, a number of non-essential proteasomal mutants were tested for their sensitivity to low levels of rapamycin. The maximum growth rate of mutant cells in YPD medium containing 50 ng ml^−1^ of rapamycin was normalized against that of the same cells grown in YPD containing the drug vehicle. The *rpn4Δ* deletion cells and the *gcn4Δ/gln3Δ* mutants (figure 7*d*) were included as negative and positive controls, respectively. When compared with WT cells, deletion of genes coding for subunits of the 19S regulatory particle (*SEM1*, *RPN1*) or the 20S core particle (*PRE9*), or genes involved in proteasome maturation and assembly (*ECM29*, *POC4, UMP1*), ubiquitin conjugation (*UFD4*), or ubiquitin synthesis and recycling (*UBI4*, *DOA4*, *UBP6*) rendered cells more sensitive to rapamycin (figure 8*a*; *p* < 0.01, *t*-test).

Pre9 is the only non-essential subunit of the 20S CP. Hypersensitivity of *pre9Δ*, *rpn1Δ*, *doa4Δ* and *sem1Δ* cells to low levels of rapamycin was reported from previous genome-wide studies [48,49]. By contrast, deletion of *RPN10* (coding for a non-ATPase base subunit of the 19S RP) led to rapamycin hyposensitivity (figure 8*a*). Similar tests were conducted on available mutants of essential proteasomal genes using DAmP strains [23]. In DAmP strains, the mRNA abundance of essential genes is typically reduced by two- to ten-fold [50]. Compared with the wild-type cells, mutants bearing hypomorphic alleles of *RPT2*, *RPN8* or *RPN11* (coding for components of the 19S RP) displayed rapamycin hyposensitivity (figure 8*b*). Conversely, rapamycin hypersensitivity was observed in cells with reduced levels of 19S subunits (Rpn3, Rpt5, Rpn7 and Rpn12), 20S components (Pup1, Pre2, Pre3, Pre5 and Pre7) or Ufd1, a subunit of the Cdc48–Npl4–Ufd1 complex responsible for recruiting polyubiquitinated proteins to the proteasome (figure 8*b*). Pup1, Pre2 and Pre3 are the three catalytically active subunits of the 26S proteasome, providing the trypsin-like, chymotrypsin-like and caspase-like activities, respectively [51–53]. The three DAmP proteasomal mutants were subjected to rapamycin dosage response assays (figure 8*c*). When there is no rapamycin in the medium, the *PUP1*^DAmP^ and *PRE3*^DAmP^ mutants grew more slowly than the wild-type cells, whereas the *PRE2*^DAmP^ mutants displayed a similar growth rate as the WT. The relative growth rates of the mutants (to the WT) decreased rapidly with increasing rapamycin concentrations (up to 15 ng ml^−1^; figure 8*c*). A further significant change of relative growth rate was only observed for the *PUP1*^DAmP^ mutant cells when the rapamycin concentration was increased above 15 ng ml^−1^ (figure 8*c*). These data confirmed that the growth (rate) of yeast cells is synergistically regulated by the functions of TOR and the proteasome, and that the core functions of the 26S proteasome were essential for yeast cells to adapt to reduced TORC1 activity. Interestingly, the three catalytically compromised DAmP mutants displayed a much faster cell growth than the wild-type cells when treated with 2 µg ml^−1^ of cycloheximide (electronic supplementary material S4), indicating that cells with reduced proteasomal activities respond differently to TOR inhibition than to translation inhibition. Faster growth of these proteasomal mutants in the presence of cycloheximide has been reported before [54], and the underlying mechanisms remain to be investigated.

## 5. Discussion

Appropriate regulation of gene transcription is vitally important for cells to both grow when conditions are favourable and to survive when exposed to stressful conditions, including nutrient starvation. The TOR signalling pathway controls cell growth by stimulating anabolic processes and suppressing a variety of stress response programmes [29]. In this study, we have shown that the proteasome regulates a significant portion of the yeast transcriptome synergistically with the TOR signalling pathway. Transcriptional synergy between the proteasome and TORC1 was confirmed by their unidirectional regulation of starvation-specific gene expression and by their cooperative actions in determining the transcription of the proteasome genes. The synergistic effects of the two pathways are also exhibited by their collaboration in cell growth control. Recently, a multi-laboratory systems biology study integrated ‘Omics’ data analysis on two laboratory yeast strains, CEN.PK 113-7D and YSBN2, and indicated that a higher rate of protein turnover and higher proteasomal activity in CEN.PK cells may account for their faster growth [55]. This observation was recently confirmed [56]. Our findings that the function of the proteasome acts synergistically with that of TOR to promote the expression of anabolic genes involved in the *de novo* biosynthesis of purines and amino acids (table 1) and to restrict the starvation and stress response (figure 2) could provide a further mechanistic explanation for this difference (figure 8*d*). The combination of TORC1 and proteasome inhibitors was shown to act synergistically to cause cell death in pre-B acute lymphocytic leukaemia [57] and to inhibit cell growth in human oesophageal adenocarcinoma [58], suggesting that the cooperative nature of the two pathways in controlling cell growth may be conserved from yeast to mammals.

Several lines of evidence suggest that Hsf1 may cooperate with Msn2/4 and Gis1 to regulate gene expression in the starvation-induced stress response (figure 8*d*). First, moderate levels of *HSP26* and *SSA3* transcripts were detected in the *msn2/4*Δ*gis1*Δ cells treated with rapamycin and MG132 (figure 5*c*). Besides STRE and PDS motifs, the heat shock element is also enriched in the promoters of class 1 genes (table 1 and figure 3*b*), and is present in the promoters of both *HSP26* and *SSA3*. Second, the Hsf1 and Msn2/4 TFs were shown to cooperate in regulating the expression of *HSP26* in starvation and stress conditions [59], and the three classes of stress response TFs (Msn2/4, Gis1 and Hsf1) were commonly activated in the long-lived *sch9Δ*, *ras2Δ* and *tor1Δ* mutants [60]. Previous studies by our group and others indicated that Msn2 and Gis1 are targets of the proteasome [7,8,61]. Recently, Hu & Mivechi [62] revealed that the mammalian homologue of Hsf1, the main regulator of the heat shock response in mammals, is degraded by the proteasome, indicating that proteasome-mediated degradation of the stress response TFs may be a common mechanism adopted by cells to reduce the severity of the stress response. By contrast, TORC1 promotes nuclear export of Msn2 [63] through the Tap42-PP2A signalling branch [64,65]. The Rim15 kinase, shown to coordinate Msn2/4- and Gis1-mediated transcription with post-transcriptional mRNA protection [32,66,67], is retained in the cytoplasm by TORC1 activity via Sch9 and 14-3-3 proteins [68,69]. Similarly, cytoplasmic retention of Yak1 is mediated by the yeast 14-3-3 protein, Bmh1 [45]. Our transcript analysis indicated that Yak1 and Rim15 act in parallel pathways to promote transcription mediated by Msn2/4 and Gis1 (figure 6). These data support the hypothesis that while the proteasome restricts starvation and stress response by controlling the levels of the stress response TFs, the TOR signalling pathway, via different downstream signalling branches, negatively modulates a number of regulators (Rim15, Yak1 and others) by retaining them in the cytoplasm. Upon TORC1 inhibition, these regulators translocate into the nucleus and cooperate to activate the starvation-induced transcription programme mediated by the Msn2/4, Gis1 and possibly Hsf1 proteins (figure 8*d*). The fidelity of the starvation-induced transcription programme requires the proteasome function to prevent activation of starvation-specific genes in exponentially growing cells (figures 1*b* and 5*a*) and to avoid excessive activation of stress response genes in TORC1-inhibited cells (figures 1*b* and 6*b*), in order to ensure optimum cell growth (figures 6*c* and 7*c*) and possibly a speedy return to exponential growth when starved cells are refed with nutrients [70].

Transcriptional activation of proteasomal genes is mediated by Rpn4 in TORC1-inhibited cells (figure 7*a*,*b*). Rpn4 is a short-lived protein and is degraded by the proteasome [31], thus providing a negative feedback loop to determine the proteasome abundance in the cell [71,72]. Loss of *RPN4* causes slower cell growth, hypersensitivity to rapamycin (figure 7*c*,*d*) and decreased cell viability when exposed to alkylating agents, arsenic, UV, DTT or cadmium [73–75], suggesting that upregulation of proteasome abundance through Rpn4 is a general mechanism adopted by yeast cells to adapt to starvation and stress. Interestingly, transcription of *RPN4* itself is regulated by a range of stress response TFs, including Hsf1, the multi-drug-resistance-related factors Pdr1 and Pdr3, and Yap1, a TF essential for response to oxidation, toxic metals and MMS [75–77]. Transcription of *HSF1*, *YAP1* and *RPN4* is significantly increased by rapamycin treatment (see electronic supplementary material S1), suggesting that Rpn4-mediated transcriptional increase in proteasomal genes may, at least partially, result from activation of the stress response network in TORC1-inhibited cells (figure 8*d*).

We have demonstrated that the proteasome and TOR pathways converge on Msn2/4, Gis1 and Rpn4 to regulate starvation-induced gene expression. How the functions of the two pathways cooperate to coordinately regulate the transcription of the ADE and amino acid biosynthetic genes (class 3, figure 2*a*) or the NCR-sensitive genes in a complementary manner (class 2, figure 2*a*) is less well understood. Over-represented motifs identified in the promoter regions of these genes suggest that the two pathways may converge on Gcn4/Bas1/Mbp1 and Gln3/Gat1, respectively, to modulate their expression (table 1). MG132 treatment leads to transcriptional downregulation of both set of genes, indicating that the proteasome function is necessary for the basal level of their expression. Proteasome-mediated degradation of Gcn4 is required for expression of Gcn4-activated genes [11]. Whether the function of the proteasome is needed for the expression of Gcn4-repressed genes [28] is not known. Equally, it cannot be ruled out that the proteasome may associate with chromatin to influence their transcription, as demonstrated for Spt23- and Mga2-regulated genes involved in lipid metabolism [18].

Connections between the UPS and the TOR signalling pathway were reported previously. Chotechuang *et al.* [78] observed that downregulation of the UPS system induced by high-protein diet requires the inhibition of AMPK and the activation of the mTOR pathways. Furthermore, the inhibition of the proteasome function represses mTOR signalling and protein translation in colon cancer cells [79]. Recently, DEPTOR, the endogenous inhibitor of mTORC1 and mTORC2, was shown to be degraded by the proteasome [80,81], mediated by the SCF^βTrCP^ E3 ubiquitin ligase [82–84], indicating that the proteasome is directly involved in modulating the function of mTOR. Although there is no *Saccharomyces cerevisiae* homologue for the mammalian DEPTOR protein, these and our own studies have provided a platform for future investigations of the complex interactions between the proteasome and TOR pathways in gene expression, cell growth and the stress response. As rapamycin does not inhibit all the functions of TOR [85], the combination of the proteasome inhibitors with TOR active-site inhibitors [86], ATP-competitive inhibitors [87] or nitrogen starvation should be included to further interrogate the complex relationship between the two pathways.

## 6. Acknowledgements

We thank Dr Pinar Pir and Dr Leo Zeef for their help with the initial transcriptome data analysis. Z.Q. thanks the Cambridge Overseas Trust and Lucy Cavendish College for financial support. This work was also supported by a BBSRC grant (no. BB/C505140/2) and a UNICELLSYS grant awarded to S.G.O. by the EC.

## Supplementary Material

Supplementary File 1

## Supplementary Material

Supplementary File 2

## Supplementary Material

Supplementary File 3

## Supplementary Material

Supplementary File 4
