# Supplementary material for: Synergistic effects of TOR and proteasome pathways on the yeast transcriptome and cell growth
Source: Open Biol. 2013 May;3(5):120137. doi: 10.1098/rsob.120137 (PMC3866871; doi:10.1098/rsob.120137)
Supplement: Supplementary File 4 [file rsob120137-s4.pptx]

## Slide 1
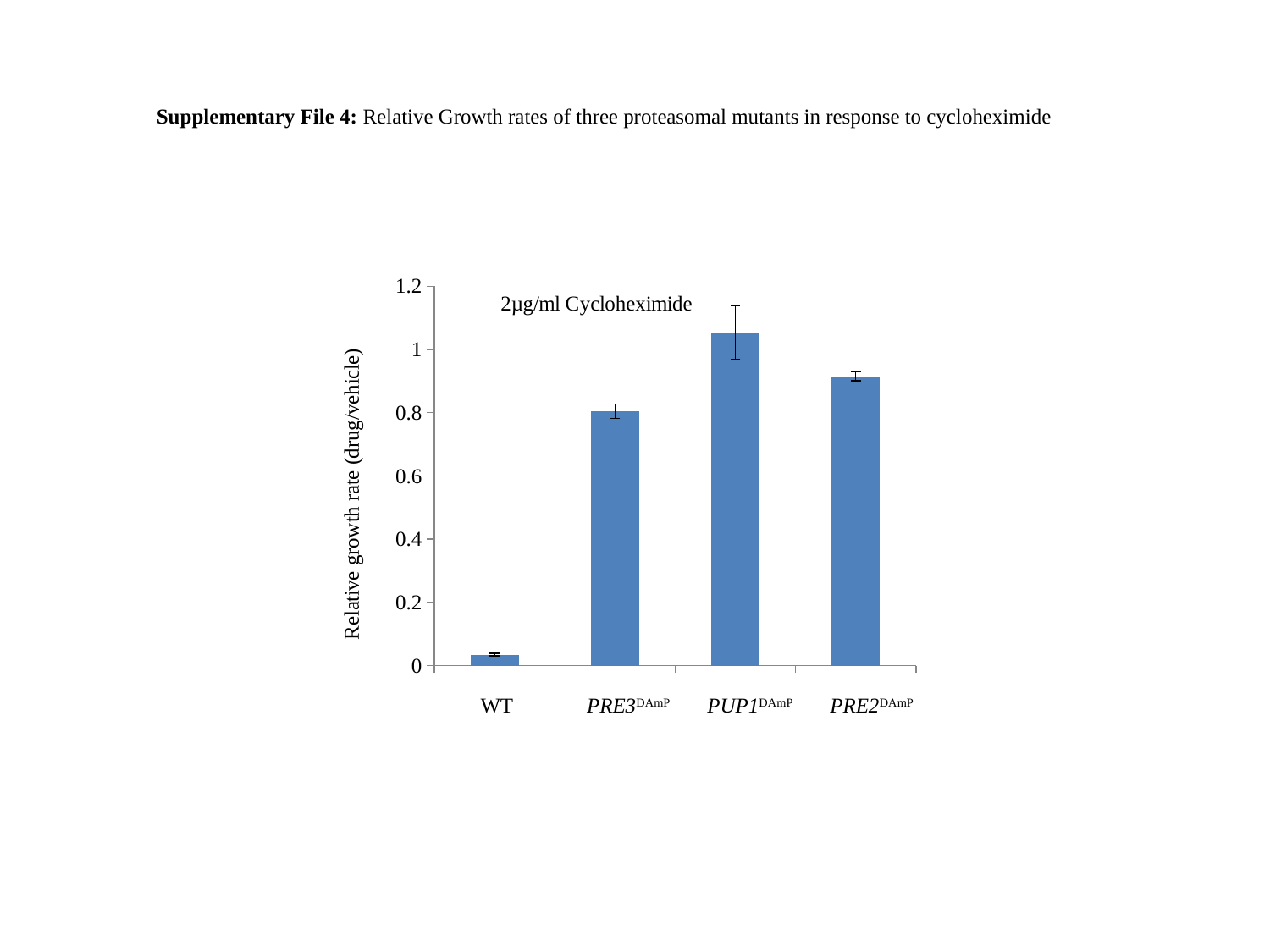

Supplementary File 4: Relative Growth rates of three proteasomal mutants in response to cycloheximide
### Chart: 2µg/ml Cycloheximide
| Category | |
|---|---|
| WT | 0.0347721020129304 |
| PRE3 (DAmP) | 0.80472716206699 |
| PUP1 (DAmP) | 1.0544736513076898 |
| PRE2 (DAmP) | 0.9153108643192991 |WT PRE3DAmP PUP1DAmP PRE2DAmP
